# Improving the Effectiveness of Electronic Health Record-Based Referral Processes

**DOI:** 10.1186/1472-6947-12-107

**Published:** 2012-09-13

**Authors:** Adol Esquivel, Dean F Sittig, Daniel R Murphy, Hardeep Singh

**Affiliations:** 1 Department of Clinical Effectiveness and Performance Measurement, St. Luke’s Episcopal Health System, Houston, TX; 2 University of Texas School of Biomedical Informatics and the UT-Memorial Hermann Center for Healthcare Quality & Safety, Houston, TX, USA; 3 Houston VA HSR&D Center of Excellence and The Center of Inquiry to Improve Outpatient Safety Through Effective Electronic Communication, both at the Michael E. DeBakey Veterans Affairs Medical Center and the Section of Health Services Research, Department of Medicine, Baylor College of Medicine, VA Medical Center (152), 2002 Holcombe Blvd, Houston 77030, TX, USA

## Abstract

Electronic health records are increasingly being used to facilitate referral communication in the outpatient setting. However, despite support by technology, referral communication between primary care providers and specialists is often unsatisfactory and is unable to eliminate care delays. This may be in part due to lack of attention to how information and communication technology fits within the social environment of health care. Making electronic referral communication effective requires a multifaceted “socio-technical” approach. Using an 8-dimensional socio-technical model for health information technology as a framework, we describe ten recommendations that represent good clinical practices to design, develop, implement, improve, and monitor electronic referral communication in the outpatient setting. These recommendations were developed on the basis of our previous work, current literature, sound clinical practice, and a systems-based approach to understanding and implementing health information technology solutions. Recommendations are relevant to system designers, practicing clinicians, and other stakeholders considering use of electronic health records to support referral communication.

## Introduction

Outpatient referrals, defined as processes that include a transfer of responsibility for some aspect of patient’s care from a referring provider to a secondary service or provider, [1] are an important but challenging aspect of primary care practice. Successful coordination of referrals hinges upon effective and timely communication to facilitate information sharing and transfer of patient care responsibilities between outpatient providers [2-10]. However, referral communication related to both provider-provider and provider-patient interactions [3,11-14] is prone to breakdown [2,14-22]. The growing use of referral care [23] suggests the need for improving reliability and efficiency of the referral process to create a greater impact on health care quality.

In accordance with the 2009 Health Information Technology for Economic and Clinical Health Act (HITECH) and its Meaningful Use goals for effective use of electronic health records (EHRs), healthcare institutions are increasingly adopting technology to support patient care. By 2015, hospitals are expected to demonstrate, among other things, the capability to exchange key clinical information among providers of care and other patient-authorized entities electronically [24]. This increasing adoption of health information technology holds promise for improving referral communication in health care [25-28]. However, early adopters of these technologies, mostly large integrated systems, have encountered novel communication challenges and unintended consequences that are important to understand in order to reduce future care delays [18,29-35].

Many referrals between primary care providers (PCPs) and specialists do not take place within the same practice or institution; and in general, providers don’t have access to the same EHR. However, efforts to address communication challenges using EHRs will be essential given the emphasis on coordination of care and exchange of relevant clinical information by the Patient Protection and Affordable Care Act of 2010 [36]. Recent reform initiatives call for healthcare institutions to become Accountable Care Organizations (ACOs) [37] and demonstrate the use of evidence-based medicine and the application of evolving technologies to support a strong foundation for coordinated primary care. They also create an expectation of continuous process improvement based on measurement of clinical quality and outcomes [38]. EHR-based referrals thus would be an essential component of patient care through ACOs. Even when supported by technology, referral communication between PCPs and specialists is often unsatisfactory [39]. This might be partially due to lack of attention on how communication technology fits with the social environment in which it is implemented [40,41]. Addressing these key challenges in making electronic referral communication effective [11,12,42] requires a multifaceted “socio-technical” approach [43].

Although efforts have been made to improve and standardize overall EHR usability, [44,45] there are presently no standards that specifically address the design or use of electronic systems in outpatient referral communication, and best practices in this area are limited [6,19,39,46-48]. In fact, no available turn-key EHR system can fully support the complexities of most referral processes. Furthermore, referral processes are highly variable across health care settings, and EHRs that support referrals are often heavily customized to reflect unique organizational requirements [19,49,50]. Although complete standardization of referral practices is neither possible nor desirable, several aspects of referral communication are amenable to strategies to reduce the risk of unintended consequences and delays in patient care.

This article describes ten recommendations that represent potential best practices to design, develop, implement, improve, and monitor electronic outpatient referral communication. Recommendations are grounded in a socio-technical model for health information technology [43]. This model uses 8 interrelated dimensions to identify challenges related to developing, implementing, and using information technology within health care (hardware & software, clinical content, human-computer interface, people, workflow & communication, organizational features, external rules and regulations, and measurement & monitoring). The recommendations are also based on current literature, sound clinical practice, our previous work, and a systems-based approach to understanding and implementing health information technology solutions. We also categorized recommendations according to the dimensions of the socio-technical model with which they are most closely related (Table 1). Some recommendations have an established evidence-base and others are based on our experiences or perspectives, but most are not widely adopted by institutions and/or current EHRs. Thus, we believe these recommendations are relevant to all system designers, practicing clinicians, and other stakeholders considering the use of EHRs to support referral communication. 

**Table 1:** Recommendations Summary and their relation to Socio-Technical dimensions

**Recommendation**		Primary Socio-Technical Dimension*
1	Include real-time clinician-to-clinician communication features as part of the referral system.	Hardware & Software
2	Design and use electronic standardized referral templates that include both structured and free-text fields.	Human-Computer Interface
3	Enforce electronic capture of the reason for the referral.	Clinical Content
4	Bring PCPs and specialists together to collaboratively develop referral guidelines for inclusion into the electronic referral system	People
5	Integrate patient communication into the electronic referral process	People
6	Use automation to pre-populate electronic referral requests with patient-specific data	Workflow & Communication
7	Include the capability of electronic consultations (information-only referrals).	Workflow & Communication
8	Close the communication loop by providing referral status tracking and feedback capabilities and integrating these tools into providers’ workflows	Workflow & Communication
9	Standardize and maintain up-to-date institutional policies and procedures for electronic referrals.	Organization Policies & Procedures
10	Monitor electronic referral communication performance.	Measurement & Monitoring

*Although recommendation may be associated with more than one dimension of the socio-technical mode, this table identifies the dimension each recommendation most directly relates to.

## Recommendation #1: Include real-time clinician-to-clinician communication features as part of the referral system

Providers often prefer traditional face-to-face or synchronous communication, such as telephone conversations. While excessive reliance on the EHR and other health information technology may diminish the use of real-time communication, certain critical situations require the interactivity afforded by direct conversation. In fact, some estimates propose that up to 60% of providers’ time in clinic is devoted to synchronous conversation [51]. In some cases, such as when a referral is urgent, real-time communication may be required to expedite the referral process [52,53]. Specialists may also want to speak directly to referring providers if there is any doubt about a referral’s appropriateness or urgency, even when PCPs and specialists share access to the patient’s record. EHRs can facilitate real-time phone conversations or internet-based audio-, video-, or text-based conferencing interactions by providing easily accessible and updated contact information for specialists and PCPs (or their clinics) on the referral interface [54-57]. This flexibility should be specified in any policies and procedures governing outpatient referrals [58].

## Recommendation #2: Design and use standardized electronic referral templates that include both structured and free-text fields

The content, form, and style of referral letters influences the referral process [5,16,59-61]. Several studies have shown increased provider satisfaction and more consistent and timely feedback from specialists when referral templates are used to standardize referral communication [16,62,63]. Electronic systems provide an excellent opportunity to create, maintain, and disseminate the use of standardized templates [64,65]. However, the interface of electronic referral templates should be designed to avoid excessive constraints that can limit providers’ ability to explain and document relevant findings [4]. Thus, when designing electronic referral templates, human-computer interface designers must maintain a delicate balance between structured fields to capture required essential information and free-text fields to allow providers to qualify and expand on their findings freely.

## Recommendation #3: Enforce electronic capture of the reason for the referral

More than fifty years ago, Williams et al. determined that providing a clear reason for a referral was an essential step in the outpatient referral process [13]. Since then, multiple studies have shown that providers’ failure to clearly state the reason for referral (a problem identified in 20-88% of referrals [7,8,21,50]) remains a major barrier to effective referral communication [20,66]. The inclusion of a clear reason to justify a referral is not only regarded as good professional practice but it has also been shown to expedite the referral process [2,7,22,67]. Therefore, electronic systems should be designed to prevent referrals from being transmitted unless they have a clearly defined reason to justify them. In addition to a standard set of generic choices, such as those proposed by Forrest et al. (to seek advice, to request a technical procedure, and to request co-management of the patient), electronic systems should give providers the option to expand and elaborate on their selection when needed [68].

## Recommendation #4: Bring PCPs and specialists together to collaboratively develop referral guidelines for inclusion into the electronic referral system

EHRs offer a robust platform for integrating referral guidelines into providers’ workflows at the point of care, and referral guidelines can improve the referral process in several ways. For instance, they can help providers determine the appropriateness of a referral prior to initiating the request [42,47] or allow a provider to anticipate the specialist’s referral information and patient work-up needs, improving efficiency and quality. People comprise one of the key dimensions of the socio-technical model. While EHRs are valuable delivery vehicles for referral guidelines, effective outcomes will only be achieved by collaborative efforts between referring providers and specialists to facilitate communication, decrease referral denials, and clarify referral expectations. While collaboration across different practice settings and institutions will be challenging to operationalize, it must also be encouraged keeping in line with the national focus on reducing health care costs and overutilization [19,69]. For instance, solo practitioners and small independent practices lacking formal organizational structures can leverage their existing networks of specialists to develop mutually agreed-upon referral guidelines. Additionally, third parties involved in regulatory, reimbursement, or quality improvement activities (e.g., regional extension centers, payers, or medical societies) can facilitate the development and dissemination of a basic set of guidelines as a starting point. Service agreements between PCPs and specialists that include referral guidelines can facilitate provider access to specialists and reduce inappropriate referrals by suggesting evidence-based pathways or alternatives to referrals [70-73]. However, given the complexity of some referrals, systems should remain sufficiently flexible to allow providers to bypass guidelines and submit a referral request that may not appear to adhere to guideline criteria by appropriately justifying its urgency and clinical need.

## Recommendation #5: Integrate patient communication into the electronic referral process

As early as 1971, researchers pointed out that the success of outpatient referrals was related in part to patient-related variables, [2] such as patient’s illness and socioeconomic background. However, subsequent work has paid little attention to the patient’s role in outpatient referral communication. In recent years, the growth of personal health records and other consumer electronic communication tools have modernized and fundamentally transformed patient-provider communication [74]. Nevertheless, patient-related communication remains vulnerable to breakdowns. For instance, these communication failures can account for a substantial number of incomplete referrals resulting in missed appointments and delays in care [53,75]. Attributes similar to those expected of provider-to-provider electronic communication (i.e., secure, timely, reliable, and actionable) [76] must also be used to inform tools to enhance patient-centered communication [77]. These attributes should be the hallmark of effective electronic communication within the patient-centered medical home model [78-80]. Hence, EHRs aimed at supporting referral communication should include functionality to allow the patient to provide additional information if and when needed, and to permit patients to become an active decision-maker during the referral process (i.e. allow them to schedule and cancel appointments, select providers, ask questions). Given the low adoption and use of existing patient communication tools [81,82], novel methods beyond traditional web-based portals are needed. System developers and administrators should explore how to leverage technologies such as smart phone apps, social media portals, and electronic outreach programs [83,84] as well as consider alternative forms of patient access or outreach in order to make patient communication more reliable. This will enable patients to have secure and timely access to relevant information such as referral status updates, reminders to increase patient compliance, and tools to facilitate communication with their physician.

## Recommendation #6: Use automation to pre-populate electronic referral requests with patient-specific data

If used appropriately, electronic referrals have the potential to enhance provider workflow by automating certain tedious or repetitive steps where manual effort is unnecessary. The cognitive load imposed by the use of structured templates, referral guidelines, and use of computerized interfaces increases the time commitment and complexity of initiating and managing referrals [85]. In a recent study, referring PCPs and specialists both suggested the use of automation to pre-populate electronic referral requests in order to decrease both workload and cognitive load [9]. In a separate study, auto-population was commended by providers as a mechanism to improve the efficiency of the consultation process [86]. Electronic referrals should harness the benefits of EHR data and use it to automatically pre-populate fields in the referral template whenever possible (e.g., demographic data, current medication list, recent relevant laboratory test results [18]). Ultimately, more advanced EHRs could even use rule-based pre-population to supply additional relevant information based on the patient’s diagnosis or age group.

## Recommendation #7: Include the capability of electronic consultations (information-only referrals)

The conceptual definition of “referral” implies an actual transfer of responsibility for some aspect of the patient’s care and an encounter with another provider. In contrast, a strict consultation involves seeking a colleague’s opinion about a particular aspect of the care of the patient, but at no time is the patient under the direct care of the consultant [1,87]. For example, certain referral questions are addressed more efficiently through consultation or information exchanges between the referring PCP and the specialist, which does not necessarily require a physical encounter between the patient and the specialist [9]. Workflow efficiency might be improved if electronic consultations are effectively used. Electronic health records can facilitate these consultations through more flexible and efficient electronic consultation processes that minimize delays (i.e., “information-only” referrals that do not require a patient visit). A successful example of this practice is the established telemedicine modality known as “store-and-forward” in which the provider exchanges relevant patient information with the consultant asynchronously and requests his or her opinion electronically [88,89]. These strategies, if implemented appropriately, can also minimize delays and inefficiencies in care related to unnecessary referrals [48].

## Recommendation #8: Close the communication loop by providing and integrating referral status tracking and feedback capabilities into providers’ workflows

Coordination of care is more effective when all interested parties are aware of the status of the referral request. Referring providers should receive timely feedback from the specialists upon denial, approval, or completion of each referral [9]. However, studies suggest that specialists fail to provide feedback in 15-45% of referrals [4,7,22,61]. Similarly, specialists may need to discuss requests with the referring providers before or after approving them. In engineering, a closed-loop control system is one in which feedback is needed to control the states or outputs of a dynamic system [90]. Often used in decision support systems, [91,92] closed-loop control can improve electronic referrals by ensuring that communication is coupled with timely and appropriate feedback. Effectively closing the loop on all outpatient referral communication requires considerable resources and efforts from all stakeholders; however, EHRs can help to close the referral communication loop in multiple ways. For example allowing providers to document and access each other’s notes about encounters, orders, and other relevant information, or by automatically notifying providers of changes in the status of the referral as it progresses through the referral stages. Additionally, the EHR can notify the referring provider when the specialist has reviewed, approved, or denied a referral request or has asked for additional information [86]. These tools must integrate into providers’ workflow in order to leverage improvements in reliability and efficiency. Nevertheless, as with other types of electronic communication in healthcare, it is important not to overload providers with excessive notifications about status updates [93]. Thus, while electronic referral communication must be comprehensive, it should be implemented in a non-intrusive manner so that information remains available to providers and patients on demand.

## Recommendation #9: Standardize and maintain up-to-date institutional policies and procedures for electronic referrals

Within institutions, lack of clear policies and procedures can result in unnecessary heterogeneity across referral processes causing inefficiencies in patient care, provider dissatisfaction, and potential for delays in diagnosis and treatment [9]. Even when organizations develop policies and procedures governing referrals, the adoption of health information technology often translates into profound changes in performance and culture [94,95]. Organizations must carefully review and continuously update policies and procedures related to referrals to ensure they reflect appropriate use of electronic tools [40]. Referral policies and procedures should provide detailed guidance with respect to every facet of the use of technology supporting the referral process. For example, to assure compliance and effective use of health information technology for referrals, organizations need to have clearly documented roles and responsibilities for PCPs, specialists, and supporting staff during key stages of the referral process. Additionally, referral policies and procedures should outline the minimum information PCPs should include in the electronic referral request, as well as expected turnaround times for specialists to respond to the referral. They should also incorporate details about the tools available to providers to monitor timeliness and effectiveness of electronic referral communication [19]. Finally, they should allow the flexibility to account for different levels of urgency and importance across clinical problems and specialties, permitting providers to expedite a particular referral when necessary [40,52,96,97]. A clear and common understanding of referral processes with documented policies and procedures of how the technology should be used by PCPs, specialists, and supporting staff is essential for success.

## Recommendation #10: Monitor electronic referral communication performance

Recent literature has revealed several serious health information technology-related errors that arose from faulty system design, configuration, or implementation processes [98-101]. Organizations must continuously monitor and evaluate the usability, performance, benefits, and drawbacks of their electronic referral systems [40]. As with any health information technology-related process, referral communication should be monitored and revised, as needed, [43] to ensure that all stakeholders’ needs are being met in a safe and efficient manner. For instance, in our previous work we found that about 7% of electronic referrals at our institution had no follow-up action by specialists at 30 days [29]. Continuous monitoring and frequent assessments of several process measurements (e.g., completed referrals, no-shows/missed appointments, and denied or cancelled referrals) should be part of the organization’s ongoing efforts to ensure the effectiveness of their electronic referral communication practices.

## Conclusion

EHR-based referrals offer the possibility of greatly improving existing outpatient referral processes. However, technology-facilitated referral processes have not yet reached their potential and will soon be put to the test given the rapid adoption of EHRs. Our proposed recommendations highlight the need to consider the socio-technical context in which information technology-based tools are implemented. Allowing for some flexibility in the referral process and monitoring communication outcomes are vital to effective implementation. As healthcare organizations continue to adopt and use EHRs, the success of technology-enabled referral processes will depend on their ability to remain patient-centered and responsive to providers’ needs. The recommendations presented address key areas within seven of the eight socio-technical dimensions, all of which must be performed while adhering to external rules and regulations (e.g., HIPAA or HITECH act), as suggested by the model’s eighth dimension. We envision that these recommendations will be useful for several types of stakeholders as they move forward in designing, implementing, and improving their electronic referral systems.

## Abbreviations

EHR, Electronic Health Record; HITECH, Health Information Technology for Economic and Clinical Health Act; PCP, Primary Care Provider; ACO, Accountable Care Organization; HIPAA, Health Information Portability and Accountability Act.

## Competing interests

The author(s) declare that they have no competing interests.

## Author’s contributions

AE participated in conceptualization and drafting of the manuscript. DFS participated in conceptualization and revising of the manuscript. DRM participated in conceptualization and drafting of the manuscript. HS participated in conceptualization and revising of the manuscript.

## Pre-publication history

The pre-publication history for this paper can be accessed here:

http://www.biomedcentral.com/1472-6947/12/107/prepub
